# Identification of novel prognostic biomarkers by integrating multi-omics data in gastric cancer

**DOI:** 10.1186/s12885-021-08210-y

**Published:** 2021-04-26

**Authors:** Nannan Liu, Yun Wu, Weipeng Cheng, Yuxuan Wu, Liguo Wang, Liwei Zhuang

**Affiliations:** The Fourth Affiliated Hospital, Harbin Medical University, Harbin, 150001 Heilongjiang China

**Keywords:** Gastric cancer, Multi-omics data, Genomic variation, Prognostic efficacy, Drug response

## Abstract

**Background:**

Gastric cancer is a fatal gastrointestinal cancer with high morbidity and poor prognosis. The dismal 5-year survival rate warrants reliable biomarkers to assess and improve the prognosis of gastric cancer. Distinguishing driver mutations that are required for the cancer phenotype from passenger mutations poses a formidable challenge for cancer genomics.

**Methods:**

We integrated the multi-omics data of 293 primary gastric cancer patients from The Cancer Genome Atlas (TCGA) to identify key driver genes by establishing a prognostic model of the patients. Analyzing both copy number alteration and somatic mutation data helped us to comprehensively reveal molecular markers of genomic variation. Integrating the transcription level of genes provided a unique perspective for us to discover dysregulated factors in transcriptional regulation.

**Results:**

We comprehensively identified 31 molecular markers of genomic variation. For instance, the copy number alteration of *WASHC5* (also known as *KIAA0196*) frequently occurred in gastric cancer patients, which cannot be discovered using traditional methods based on significant mutations. Furthermore, we revealed that several dysregulation factors played a hub regulatory role in the process of biological metabolism based on dysregulation networks. Cancer hallmark and functional enrichment analysis showed that these key driver (KD) genes played a vital role in regulating programmed cell death. The drug response patterns and transcriptional signatures of KD genes reflected their clinical application value.

**Conclusions:**

These findings indicated that KD genes could serve as novel prognostic biomarkers for further research on the pathogenesis of gastric cancers. Our study elucidated a multidimensional and comprehensive genomic landscape and highlighted the molecular complexity of GC.

**Supplementary Information:**

The online version contains supplementary material available at 10.1186/s12885-021-08210-y.

## Background

Gastric cancer (GC) is the fourth most common cancer and remains the second leading cause of death of all malignancies worldwide [1, 2]. Advances in the early diagnosis and treatment of GC contribute to timely curative resection or chemotherapy for patients [3, 4]. However, the metastasis and recurrence of GC gradually develop due to tumour evolution, resulting in a poor prognosis, with a dismal 5-year survival rate of only approximately 29.3% [3, 4]. Genetic factors may play an important role in GC due to genomic variation and aberrant gene expression, leading to a malignant phenotype [5, 6].

Several major cancer sequencing projects, such as The Cancer Genome Atlas (TCGA), the International Cancer Genome Consortium (ICGC), and Therapeutically Applicable Research to Generate Effective Treatments (TARGET), have created a comprehensive catalogue of genomic variations across all major cancer types, including GC [7, 8]. Cancer genomics has produced extensive information on cancer-associated genes. In recent decades, based on the rapid development of high throughput technology, numerous biomarkers have been identified and applied to targeted treatment [9, 10], such as *HER2* (human epidermal growth factor receptor 2) in breast cancer and *EGFR* (epidermal growth factor receptor) in lung cancer [11]. Driver mutations are required for the cancer phenotype, whereas passenger mutations are irrelevant to tumour development and accumulate through DNA replication [7, 12]. Although many bioinformatics tools dedicated to driver genes identification have been developed [13, 14], the number and specificity of cancer-driver genes remain a matter of debate, thus, distinguishing driver genes from passenger genes poses a formidable challenge for cancer genomics [15].

Owing to the clinical and genetic heterogeneity of cancer, the currently screened driver genes of GCs are far from aiding in the prevention and treatment of this fatal disease. Therefore, it is critical to identify more reasonable biomarkers for assessing the response to therapy and predicting prognosis in GC patients. In this study, we integrated multi-omics data of GC from the TCGA cohort to identify prognosis-related key driver (KD) genes that drive the development and progression of GC. We revealed the biological functions of KD genes, such as programmed cell death, and the clinical characteristics, including the drug response patterns and the prognostic efficacy of expression signatures in GC patients.

## Methods

### Data source

We obtained multi-omics data of 293 primary gastric cancer patients of The Cancer Genome Atlas (TCGA) from cBioPortal data sources (https://www.cbioportal.org/), including genome-wide human SNP array 6.0 copy number alteration (level 3), IlluminaGA DNASeq mutation (level 2), IlluminaHiSeq RNASeqV2 mRNA expression (level 3), and miRNA expression (level 3), as well as clinical data of 265 patients. In addition, we acquired two datasets of gene expression profiles matched by the disease and normal samples (both sample size to exceed 20), as well as corresponding clinical data from the Gene Expression Omnibus (GEO) database (accession code were GSE13911 and GSE29272, the sample size was 69 and 268, respectively). The relationships of transcriptional factor (TF) targeting mRNA were from Transfac [16], UCSC [17], and Chipbase [18], while that of miRNA regulating mRNA was from miTarbase [19] and Starbase [20]. Based on a previous study [21], we found there were no major batch effects for the expression data or copy number data. Besides, we downloaded extra data of gastric cancer patients from the Firehose database (https://gdac.broadinstitute.org/).

### Building binary genomic variation profile of coding genes

For mRNA expression data from RNAseqV2, we performed log_2_(RESM+ 1). For DNA copy number alteration (CNA), we retained CNA gain and loss, as well as high-level amplification and homozygous deletion discretized by GISTICv2 [22]. For mutation, silent (synonymous) substitutions were discarded. We built a binary somatic CNA profile of protein coding genes, where the copy number altered was 1 and wild-type was 0. By integrating the CNA spectrum and the genomic mutations, a binary spectrum of the genomic variation of the coding genes was formed (1, 0 represents the variation and the wild type, respectively). According to previous studies [23, 24], four requirements were for candidate key genes selection. (i) gene should have a dominant CNA type (amplification or deletion, binomial test, *p* ≤ 0.05); (ii) RSEM of the gene was above 0 in more than 75% of cancer samples; (iii) gene should have concordant changes between CNA and expression, that is, copy number amplification upregulates its gene expression level, and copy number deletion downregulates expression level; (iv) to improve the accuracy of the calculation, we require the frequency of genomic variation to exceed 0.1.

### Identification of prognosis-related key driver genes

We have downloaded two datasets of matched disease and normal sample gene expression profiles from GEO (GSE13911 and GSE29272), and performed the differential expression analysis using R package DESeq2 at FDR ≤ 0.05 and *p*-value ≤0.01. Then, we obtained the intersection of the differential expression genes and the candidate key genes with genomic variation, and then used the clinical data in cBioPortal to train the clinical model. Considering the genomic variation and patient’s survival information, including overall survival (OS, defined as the time from randomization to death from any cause, is a direct measure of clinical benefit to a patient.) and disease-free survival (DFS, defined as the time from randomization to the recurrence of tumour or death, and it is typically used in the adjuvant treatment setting.), we screened for the prognostic genes whose genomic variation had an impact on patients survival (in both types of survival, OS and DFS, the minimum *p*-value was required to be less than 0.05) used log-rank test. Combining the above candidate key genes, we determined prognostic related key driver (KD) genes.

### Construction of transcriptional dysregulation network

The experimental confirmation data of miRNA-target gene regulatory relationships was from known miRNA-target interaction databases (miTarbase and Starbase), and the experimental confirmation and prediction data of the regulatory relationship between TF and target genes was from known TF-target interaction databases (Transfac, UCSC and Chipbase). Based on known knowledge of expression regulation [23], only regulatory relationships that satisfied the following requirements have remained: 1) miRNA’s expression has negative (Pearson’s correlation coefficient < 0, FDR < =0.05) regulatory effect on its target gene’s expression; 2) The relationship between TF and target gene must be significant (FDR < =0.05). Benjamini & Hochberg correction were be used among all relationships between regulators (miRNAs and TFs) and KD genes.

### Functional enrichment analysis of KD genes

A web server g:Profiler can be used for gene functional enrichment analysis, including Gene Ontology terms (like molecular function (MF), biological process (BP) and cellular component (CC)) and pathways from KEGG, Reactome and WikiPathways. This web server can also be used for gene set enrichment analysis, including miRNA targets from miRTarBase and regulatory motif matches from TRANSFAC. We inputted the KD genes list on g:Profiler web server, and used FDR < 0.05 to screen the functions and target factors (miRNAs and TFs), which were thought to be enriched by KD genes.

### Drug sensitivity analysis

Gastric cancer drug sensitivity data were generated from ongoing high-throughput screening performed by the Cancer Cell Line Encyclopedia (CCLE) from Broad Institute [25] and the Cancer Genome Project (CGP) at the Wellcome Trust Sanger Institute [26]. The values we obtained on the websites include the half-maximal inhibitory concentration (IC50), genes expression level across cancer cell lines for each experiment. We acquired 24 anticancer compounds screened across 38 gastric cancer cell lines and 251 compounds across 25 gastric cancer cell lines from CCLE and CGP, respectively. The effect measures are the spearman’s rank correlation coefficient between the drug IC50s and gene expressions (for example, an effect of − 0.5 or 0.5 indicates a decrease or increase in drug concentration, respectively).

### KD genes construction grouping signatures

Using the Pearson correlation coefficient of all KD gene expression levels, we measured the similarity of the patients with gastric cancer. Based on the matrix of the Pearson similarity, the patients were classified by using hierarchical clustering. To determine the optimal number of categories for the gastric cancer patients, we calculated the Elbow method for the number of categories from 1 to 10. The Elbow method was calculated the total within-cluster sum of square (WSS) using R function fviz_nbclust from packages “factoextra” and “NbClust”. The WSS value was consistent with the classification performance of the model.

## Results

### Identification of candidate genes driving the development of GC based on multi-omics data

**Fig. 1:**
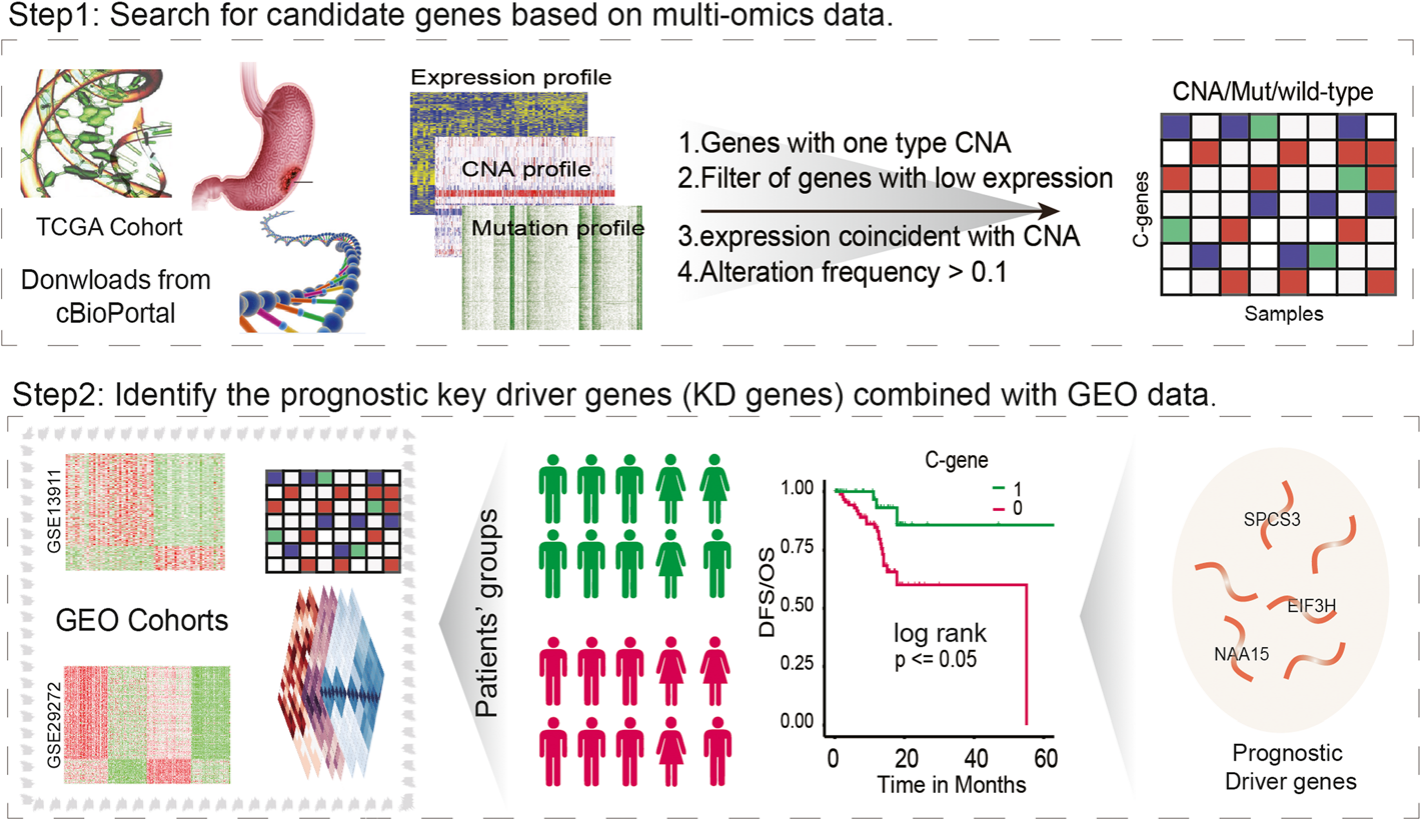
The overview. Step1, The genomic variation spectrum of gastric cancer patients were constructed using TCGA multi-omics data (including copy number alteration, mutation and mRNA expression level), and the candidate genes were screened. Step2, Identification of key driver (KD) genes related to prognosis in gastric cancer patients based on expression data and clinical data. Step3, The transcriptional dysregulation network of gastric cancer patients was constructed based on the relationship between known regulatory factors (miRNAs and TFs) and target genes

Cancer genomics has produced extensive information on cancer-associated genes [27]. Driver mutations of cancer confer tumour cell growth advantages during carcinogenesis and disease progression, however, distinguishing driver mutations from passenger mutations poses a formidable challenge for cancer genomics [7, 15]. To identify the driver events in GCs and explore their effects on tumour progression, we analysed the multi-omics data of 293 patients from the cBioPortal data resource, including genomic data (copy number alterations and somatic mutations) and transcriptome data (expression of mRNAs, miRNAs and TFs) (Table 1). We constructed a genomic variation binary spectrum of protein-coding genes in GC by pre-processing multi-omics data and integrating gene CNA, somatic mutation and gene expression data (Fig. 1).

**Table 1:** Basic information on gastric cancer multi-omics data

	CNA (SNP 6.0)	Expression (RNA-Seq)	Mutation	miRNA (RNA-Seq)	TF
Samples	293	265	289	395	265
Genes	20,558	20,132	17,172	–	–

**Fig. 2:**
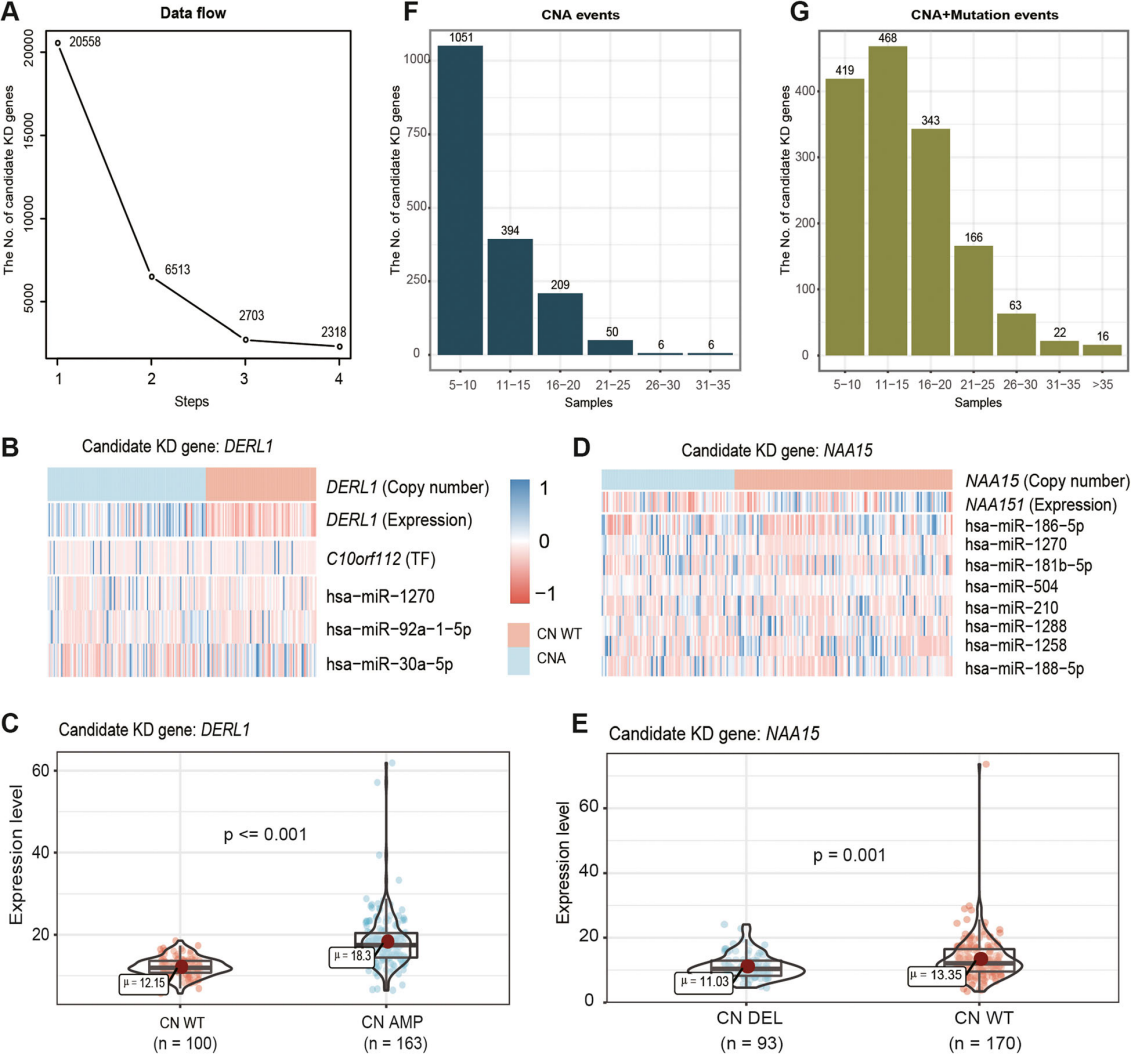
Screening genomic variation candidate genes using multi-omics data. **a**, Data flow of the candidate genes screening process. **b**, **d**, The examples show the copy number alteration of the candidate KD genes *DERL1* (**b**) and *NAA15* (**d**) on DNA and the expression level on the mRNA, as well as the expression level of its dysregulation factors. **c**, **e**, Examples showing the effects of candidate KD genes *DERL1* (**c**) and *NAA15* (**e**) copy number alteration on expression level (Wilcoxon Rank Sum Test). **f**, **g**, sample distribution of candidate KD genes, including before (**f**) and after (**g**) the addition of mutation information

After multiple steps of screening, we obtained a total of 2318 candidate genes, which potentially drove the development and progression of GCs (Fig. 1, Fig. 2a). In the process of integrating multi-omics data, we required that the candidate genes should have concordant changes between copy number alterations (CNAs) and mRNA expression. For example, the candidate gene *DERL1* was recognized to have copy number amplification, and patients with CNA of *DERL1* had significantly higher mRNA expression levels than wild-type patients (*p* < = 0.001, two-sided Wilcoxon’s rank-sum test; Fig. 2 b, c). Conversely, for the candidate gene *NAA15*, patients with copy number deletion had significantly lower mRNA expression levels than wild-type patients (*p* < = 0.001, two-sided Wilcoxon’s rank-sum test; Fig. 2d, e). As a result, the number of genes decreased rapidly as the sample size increased when the requirement that the CNA must correspondingly affect the mRNA expression itself was implemented (Fig. 2a, f). After integrating CNA data and mutation profiles, we found that the candidate genes had a high variation frequency, which ranged from 16.9 to 69.8% (the average was 39.1%), and the variant sample size of most genes was between 11 and 20 (Fig. 2g). Additionally, we found that the number of genes with more than 30 variant samples reached 38 (Fig. 2g). This suggests that adding somatic mutation information conductive to more comprehensive genomic variation research.

### Determining prognosis-related key driver (KD) genes

**Fig. 3:**
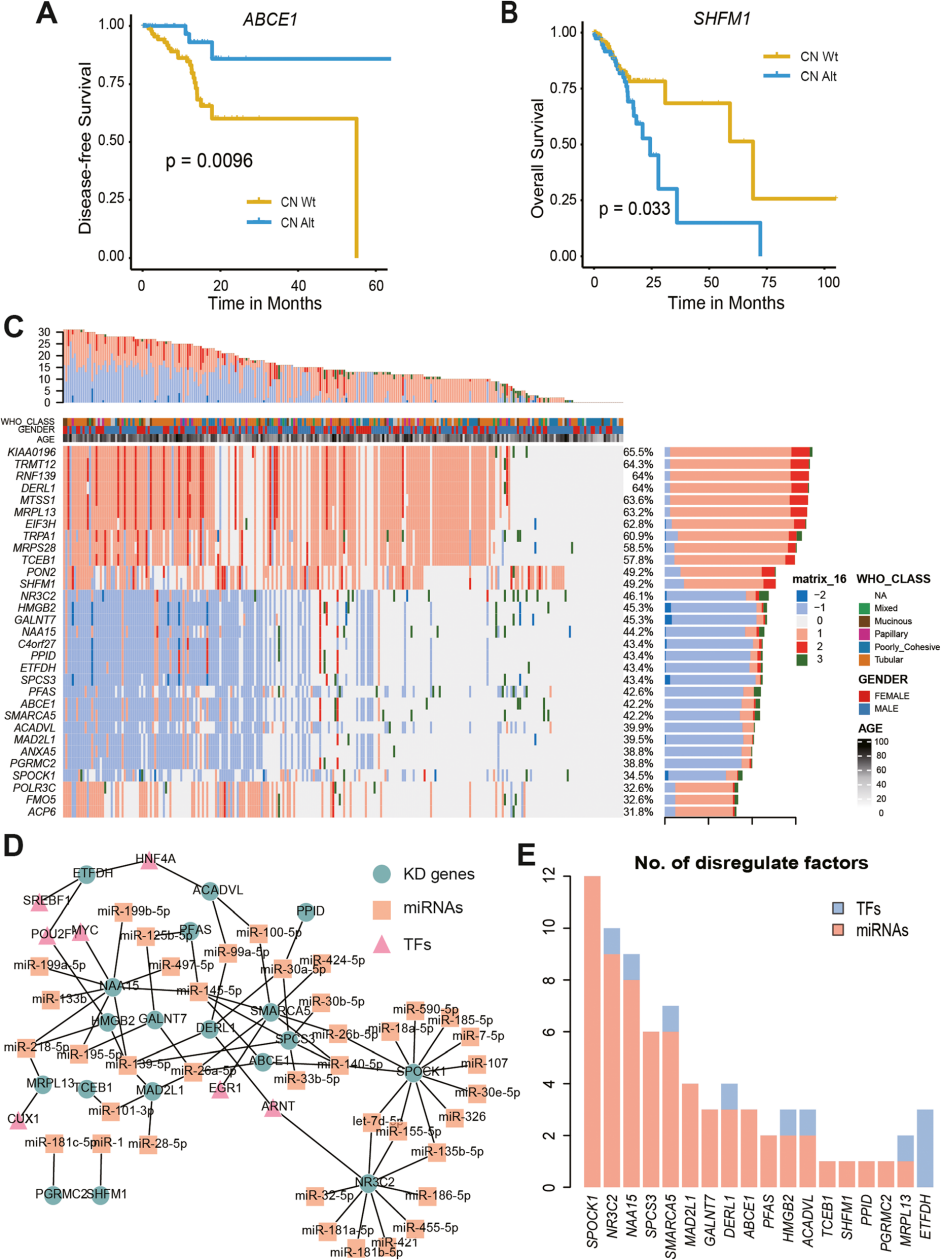
Construction of prognostic-related transcriptional dysregulation network of KD genes. AB, Examples show the prognostic efficacy of KD genes *ABCE1* (**a**) and *SHFM1* (**b**) gastric cancer patients. **c**, Genomic variation of all recognized KD genes in gastric cancer patients, including high copy number copy (dark red), low level amplification (bright red), homozygous copy number deletion (dark blue), heterozygous deletion (Bright blue), as well as somatic mutation (green). **d**, The transcriptional dysregulation network of KD genes. **e**, The number of dysregulation factors for KD genes

Studies have shown that cancer driver mutations can provide tumour cells with a growth advantage, which contributes to tumour initiation, progression, or metastasis [28, 29]. Key driver genes should have an impact on the patient’s survival time. Therefore, it is urgent to identify key driver genes from numerous tumour genome events. First, we obtained two expression profile datasets of GCs from the GEO database (GSE13911 and GSE29272), and performed gene differential expression analysis. Hierarchical clustering analysis showed that the candidate genes were disorders in GC patients compared to normal patients (Fig. S1AB). By overlapping the differentially expressed genes with the above-identified candidate genes, we obtained candidate driver genes with different expression levels in tumour tissues and normal tissues (388 in total). For each candidate driver gene, the cancer samples were split into two groups according to their copy number status (one group with CNA and the other without). Then, we used the clinical data of GC patients for survival analysis based on CNA status. Finally, we obtained a total of 31 prognosis-related key driver (KD) genes (Supplementary Table 1). For example, patients with the KD gene *ABCE1* CNA (deletion) had significantly better disease-free survival (DFS) (*p* < 0.0096, log-rank test; Fig. 3a). In contrast, patients with the KD gene *SHFM1* CNA (amplification) had significantly worse overall survival (OS) (*p* < 0.033, log-rank test; Fig. 3b). Furthermore, we mapped the genomic variation landscape of the 31 prognosis-related KD genes in GC patients (Fig. 3c). We found that these KD genes had high genomic variation frequencies (including CNAs and somatic mutations). For example, *KIAA0196* (also known as *WASHC5*) had the highest genomic variation frequency (65.5%); copy number amplifications occurred in 189 patients (where 23 patients had high-level amplifications), and somatic mutations occurred in 12 patients (Fig. 3c). In addition, the variation frequency of the KD gene *ABCE1* (ATP-binding cassette E1) was 42.2%, which included copy number deletions in 103 patients (all were copy number losses) and somatic mutations in 5 patients (Fig. 3c). *ABCE1* is a member of the ATP-binding cassette transporter family and regulates a broad range of biological functions including viral infection, cell proliferation, and anti-apoptosis. Previous research has shown that *ABCE1* plays an essential role in lung cancer progression and metastasis [30]. In our study, however, *ABCE1* was a key driver gene associated with prognosis in GC.

### Construction of transcriptional dysregulation networks of KD genes

Recent studies have confirmed that regulatory factors can affect the transcriptional activity of protein-coding genes that contribute to disease [31, 32]. Regulatory factors such as miRNAs function in RNA silencing and the posttranscriptional regulation of gene expression, which via base-pairing with complementary sequences within mRNA molecules [33]. Transcription factors that are activators can help turn specific genes “on” or “off” by binding to nearby DNA [32]. For example, the oncogenic TF *TAL1* can produce a modified autoregulatory circuitry that drives the oncogenic program in T-cell acute lymphoblastic leukaemia [32]. Thus, KD genes should have a close functional association with regulatory factors in the biological network. We obtained experimental and/or predicted miRNA-target gene pairs and TF-target gene pairs from known databases (Table 2). After calculation, we determined the regulatory factors (TFs and miRNAs) with significant regulatory coefficients for each KD gene (Fig. S2AB). Simultaneously, using the regulatory relationships between regulatory factors and KD genes, we constructed a transcriptional dysregulation network (Fig. 3d). Consistent with previous studies, we found that regulatory factors, particularly miRNAs, played a crucial role in cell growth and development [34, 35]. For example, the KD gene *SPOCK1* was regulated by 12 miRNAs, including miR-7-5p, miR-155-5p, miR-326, and miR-107 (Fig. 3d, e). Research shows that *SPOCK1* can promote the invasion and metastasis of gastric cancer through Slug-induced epithelial-mesenchymal transition [36]. MiR-155-5p can form a regulatory feedback loop with *STAT1* and might trigger cancer immunoediting to allow tumour cells to escape immunosurveillance and even to promote tumourigenesis [37]. Additionally, the KD gene *NR3C2* was regulated by 9 miRNAs including miR-7d-5p, miR-155-5p, miR-421 and miR-32-5p (Fig. 3d, e). Studies have shown that *NR3C2* plays a role in transcription regulator and molecular transducer activity [38], and can be inhibited by the migration inhibitory factor (MIF) induced signalling pathway, which is a key driver of disease aggressiveness in patients [39]. From the dysregulation network, we found that miR-7d-5p, miR-155-5p and miR-135b-5p synergistically regulated the KD genes *SPOCK1* and *NR3C2* (Fig. 3d, e). In addition, several miRNAs could simultaneously regulate multiple KD genes; for instance, miR-140-5p regulated the KD genes *SPOCK1* and *ABCE1*, and miR-26b-5p regulated *SPCS3* and *SMARCA5* (Fig. 3d). Studies have shown that miR-140-5p is a tumour suppressor in gastrointestinal cancer [40, 41]. In particular, in gastric cancer, miR-140-5p suppresses the proliferation, migration and invasion of tumour cells by regulating *YES1* [42]. This indicates that these miRNAs play a “hub” regulatory role in the process of biological metabolism and have a biological significance.

**Table 2:** Information of TF and miRNA regulating mRNA used in this study

	Interaction	Regulator	Target	Resources
TF-gene	626,331	752	19,257	Transfac [16], UCSC [17], Chipbase [18]
miRNA- gene	680,008	2562	16,339	miRTarbase [19],starbase [20]

In the dysregulation network, a total of 7 TFs were involved (Fig. 3d, e, Fig. S2B). The TF *MYC* positively regulated the KD gene *NAA15* (Padj = 9.95e-08, *R* = 0.32), *CUX1* negatively regulated *MRPL13* (Padj = 0.001, *R* = − 0.20), and *EGR1* negatively regulated *SMARCA5* (Padj = 0.022, *R* = − 0.14). Similarly, we found that multiple TFs synergistically regulated KD genes. For example, the TFs *HNF4A*, *SREBF1* and *POU2F1* simultaneously regulated the transcription of the KD gene *ETFDH*, which was upregulated by *HNF4A* (Padj = 3.5e-04, *R* = 0.22) and *SREBF1* (Padj = 0.02, *R* = 0.14), but downregulated by *POU2F1* (Padj = 7.05e-06, *R* = − 0.27) (Fig. 3d, e, Fig. S2B). Previous studies have shown that *SREBF1* (also known as *SREBP-1*) is a key regulator of fatty acid metabolism and plays a pivotal role in the transcriptional regulation of different lipogenic genes that mediate lipid synthesis, thus acting as a cancer promoter in human diseases [43, 44]. TFs are dysregulation factors of KD genes, and we also found hubs in the dysregulation network. For instance, the TF *HNF4A* regulated KD genes *ETFDH* and *ACADVL* (Padj = 3.3e-04, *R* = 0.33), and *ARNT* regulated the KD genes *NR3C2* (Padj = 3.34e-04, *R* = 0.22) and *DERL1* (Padj = 3.51e-05, *R* = 0.25) simultaneously (Fig. 3d). Wang H et al. generated a miRNA-TF regulatory network, and discovered 5 regulators that might have critical roles in colorectal cancer pathogenesis, which was helpful to understand the complex regulatory mechanisms and guide clinical treatment [45]. Interestingly, in our study, KD genes were regulated by multiple types of dysregulation factors. For example, the KD gene *ACADVL* was regulated by the TF *NHF4A* and the miRNAs miR-100-5p and miR-99a-5p, while the KD gene *RAA15* was regulated by *MYC*, miR-497-5p and miR-145-5p (Fig. 3d, e). Hao S et al. revealed that 5 miRNAs (including miR-145, miR-497, miR-30a, miR-31, and miR-20a) were considered to regulate tumour cell proliferation through TFs [46]. These findings suggest that transcriptional regulators play a crucial role in the dysregulation of KD genes, and studies of these dysregulation factors may facilitate biomarker discovery.

### Functional mechanisms of KD genes and dysregulation factors

**Fig. 4:**
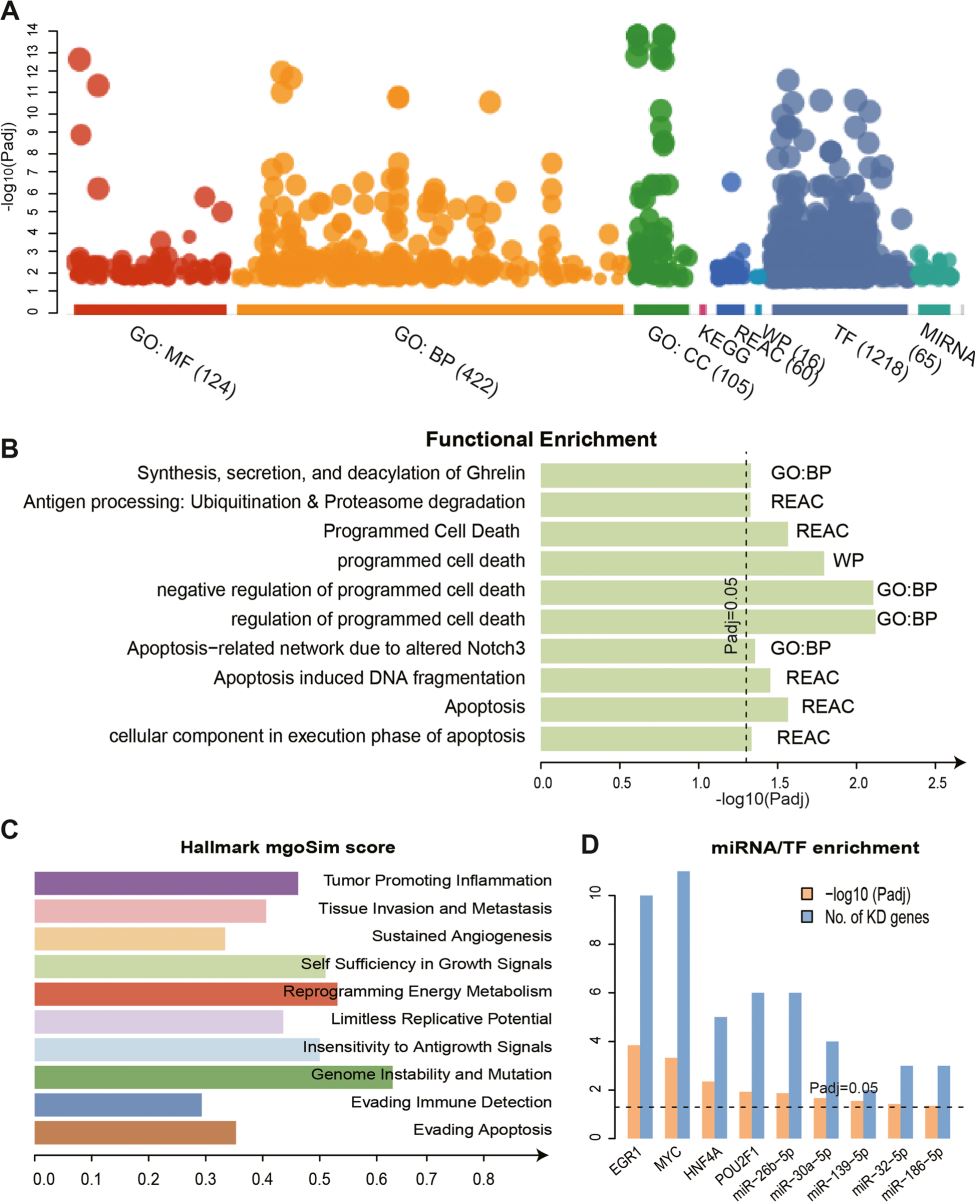
Functional enrichment analysis of KD genes. **a**, Functional enrichment analysis for KD genes using g:profiler online tool, including Gene Ontology (MF, BP, CC) and pathway (REAC and WP) in different colors. The Y axis represents -log10(Padj). **b**, The functions enriched by KD genes. The X axis represents -log10 (Padj), and the dotted line indicates Padj = 0.05. **c**, The semantic similarity between the functions enriched by KD genes and the known cancer hallmarks. **d**, The regulatory factors for KD genes confirmed by enrichment analysis. Blue indicates the number of KD genes, and orange indicates significance, −log10 (Padj)

To characterize the molecular mechanisms of the KD genes, we first used the g:profiler online tool for functional enrichment analysis. The result showed that our KD genes were enriched in many types of biological functions, including Gene Ontology MFs, BPs, and CCs, pathways (REAC and WP) (Fig. 4a). In detail, the KD genes were enriched in terms related to apoptosis-associated functions, such as “Apoptosis”, “Programmed cell death”, “Apoptosis-related network due to altered Notch3 in cancer”, and “Apoptosis-induced DNA fragmentation”. (Fig. 4b). In addition, several KD genes were enriched in terms related to immune-associated functions, such as “Antigen processing: Ubiquitination & Proteasome degradation” (Fig. 4b). To further characterize our results, we sought to characterize the cancer hallmark landscape of the KD genes. In brief, we calculated the semantic similarity score between KD genes-related GO terms and known cancer hallmark-related GO terms [47]. We found that the semantic similarity between our KD genes and the apoptotic-related cancer hallmark, “Evading Apoptosis”, was 0.35. Additionally, the semantic similarities with two immune-related cancer hallmarks (“Evading Immune Detection” and “Tumour Promoting Inflammation”) were 0.3 and 0.48, respectively (Fig. 4c). For the cancer hallmark “Genome Instability and Mutation”, the semantic similarity with KD genes was the highest, reaching 0.65 (Fig. 4c), which indicates that the genomic variation of KD genes has important functional mechanisms, including genome instability, and thus plays a carcinogenic role in biology [48]. Interestingly, our KD genes were enriched in functions related to the synthesis and secretion of gastric hormones, such as “Synthesis, secretion, and deacylation of Ghrelin” (Fig. 4c). Ghrelin is an endogenous peptide hormone mainly produced in the stomach. Previous research has shown that ghrelin can be a promising therapeutic option for cancer cachexia [49]. In addition, the KD genes were also enriched in functions related to cell growth, development and metabolism, such as “Biosynthetic process”, “Negative regulation of biological process” and “Cellular macromolecule metabolic process”.

Our above results indicate that the regulators of KD genes play an important regulatory role in carcinogenesis. Using g:profiler, we also observed that KD genes were enriched in regulatory relationships with TFs and miRNAs (Fig. 4a). To study the functional mechanisms of regulators, we used the enrichment analysis of KD genes for further characterization. By comparing the regulators, we identified the TFs and miRNAs enriched by the KD genes, and confirmed that 4 TFs and 5 miRNAs showed enrichment (Fig. 4d). The 4 confirmed TFs included *EGR1* (enrichment significance *P* = 1.42e-04), *HNF4A* (*P* = 4.42e-03), *POU2F1* (*P* = 7.67e-03) and *MYC* (*P* = 4.70e-04) (Fig. 4d; Supplementary Table S2). *EGR1* regulated 10 KD genes such as *SMARCA5*, *POLR3C*, and *MRPL13*. (Fig. 4d). The TF *HNF4A* simultaneously regulated two KD genes *ACADVL* and *ETFDH*. In addition, the TFs combination of *HNF4A* and *POU2F1* regulated the KD gene *HMGB2* (Fig. 3d; Supplementary Table S2). It is worth noting that the combination of the TF *MYC* and miR-139-5p regulated the KD gene *NAA15* (Fig. 3d; Supplementary Table S2). MiR-139-5p (*P* = 0.028) was a confirmed miRNA by enrichment that regulated two KD genes *HMGB2* and *DERL1* (Fig. 3d; Supplementary Table S2). In addition, miR-30a-5p (*P* = 0.021) regulated the KD genes *SPCS3*, *PPID* and *DERL1* (Fig. 3d; Supplementary Table S2). The miRNAs confirmed by KD gene enrichment also included miR-26b-5p (*P* = 0.013), miR-32-5p (*P* = 0.037) and miR-186-5p (*P* = 0.044) (Fig. 4d). In summary, functional enrichment analysis indicated that the KD genes were involved in vital biological functions, and further demonstrated that the regulators can be used as potential biomarkers for further experimental studies.

### Drug response effects in preclinical cell models of GC

**Fig. 5:**
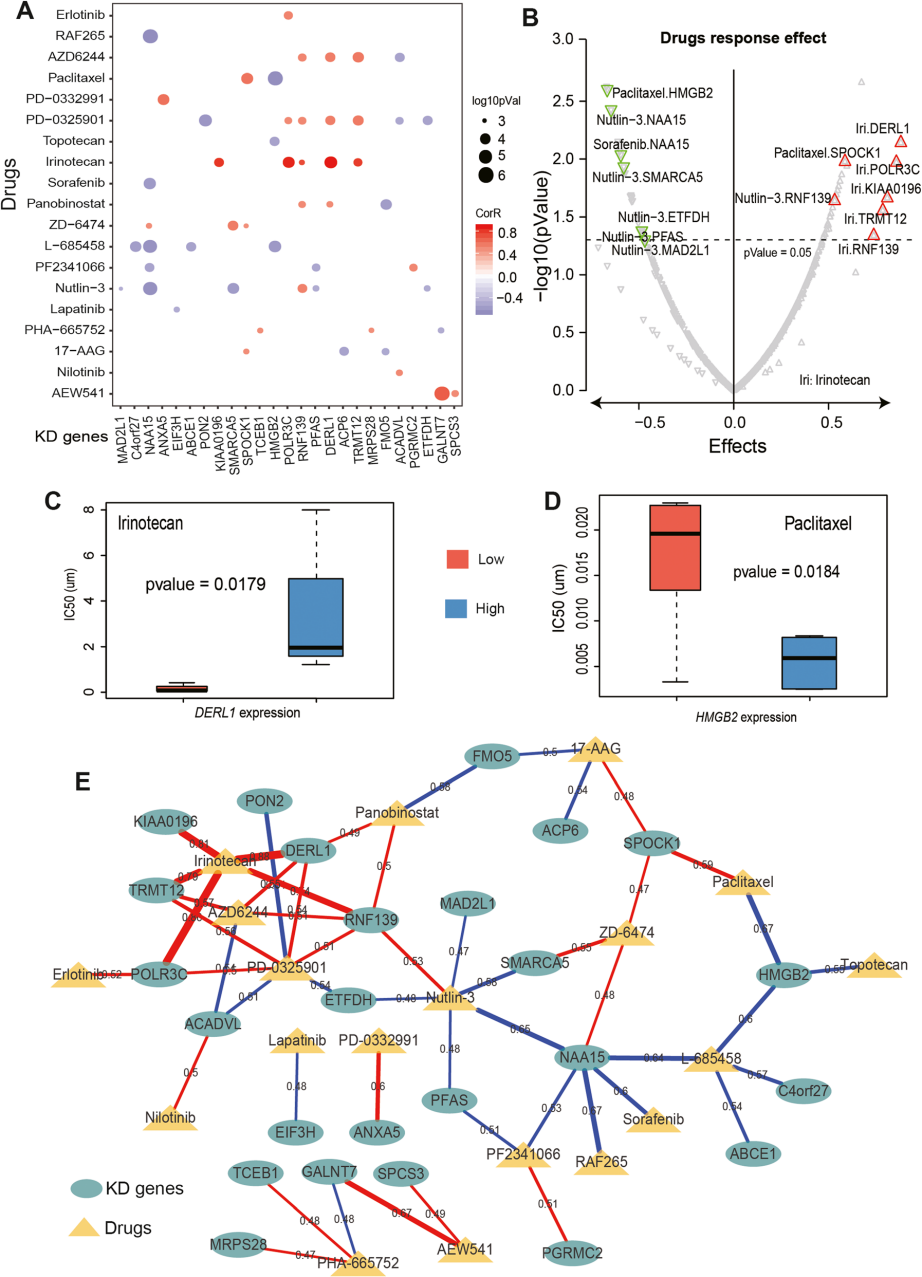
Analysis of drug response effects of KD genes. **a**, Correlation analysis between the expression level of KD genes and drug IC50 in CCLE cell model. The point size indicates the level of significance, and the color indicates the correlation coefficient, red (positive), blue (negative). Spearman’s Rank correlation. **b**, The volcano map shows the response of particular drugs, red indicates resistance, and green indicates sensitivity. **c**, **d**, The response pattern of specific drugs on KD genes, irinotecan showed drug resistance on *DERL1* (**c**), and paclitaxel showed drug sensitivity on *HMGB2* (**d**). **e**, The drug response pattern of the drugs in all KD genes in the CCLE cell model, including drug resistance (red) and sensitivity (blue), and line thickness indicates response effect

To explore the potential effects of KD genes on drug response, we evaluated whether their expression level could influence drug response across 38 preclinical cell models of GC from Cancer Cell Line Encyclopedia (CCLE). We found that multiple KD genes presented strong correlations with the drug response in GC cells (Fig. 5a). For example, irinotecan, a broad spectrum anticancer drug, showed a significant positive correlation with the expression levels of 5 KD genes in GC cells, including *KIAA0196* (*R* = 0.81, *P* = 0.02), *POLR3C* (*R* = 0.86, *P* = 0.01), *RNF139* (*R* = 0.74, *P* = 0.04), *DERL1* (*R* = 0.88, *P* = 0.007), and *TRMT12* (*R* = 0.79, *P* = 0.02) (Fig. 5a-c). This result indicated that the expression levels of these KD genes could enhance the resistance of irinotecan in GC cells. Studies have shown that patients with advanced gastric cancer are often treated with irinotecan monotherapy as salvage-line therapy [50].

Sorafenib is a multikinase inhibitor with activity against angiogenesis and the RAF-MEK-ERK pathway; it inhibited the proliferation of human gastric cancer cell line, and may reverse resistance to cisplatin by downregulating MDR1 expression [51]. In our study, however, the drug response to sorafenib in GC cells showed a significant negative correlation with the expression level of the KD gene *NAA15* (*R* = − 0.6, *P* = 0.0092) (Fig. 5a-b). Moreover, paclitaxel, a widely used anticancer drug, exhibited multiple response patterns at the expression level of the KD genes. For instance, paclitaxel showed strong resistance in GC cells with upregulated *SPOCK1* expression (*R* = 0.59, *P* = 0.01), but showed strong sensitivity in GC cells with upregulated *HMGB2* expression (*R* = − 0.67, *P* = 0.002) (Fig. 5a, b and Fig. 5d). Studies have shown that nab-paclitaxel as second-line treatment in locally advanced inoperable or metastatic gastric and gastroesophageal junction carcinoma is a promising chemotherapy regimen [52, 53].

Interestingly, the KD genes we identified have response effects with multiple anticancer drugs (Fig. 5a, e). For example, in addition to sorafenib, RAF265 (*R* = − 0.67, *P* = 0.0023), Nultlin-3 (*R* = − 0.65, *P* = 0.0037) and L-685458 (*R* = − 0.64, *P* = 0.0042) all showed strong drug sensitivity in GC cells with upregulated *NAA15* expression (Fig. 5a, e). However, the small molecule compound ZD-6474 showed significant drug resistance at the expression level of *NAA15* (*R* = 0.48, *P* = 0.045). In addition to irinotecan, multiple drugs showed strong resistance at the expression level of the KD gene *DERL1*. For instance, the responses to drugs, including AZD6244 (*R* = 0.55, *P* = 0.019), PD-0325901 (*R* = 0.54, *P* = 0.020) and panobinostat (*R* = 0.49, *P* = 0.041), presented a significant positive correlation with *DERL1* (Fig. 5a, e). A study has shown that the pan-histone deacetylase inhibitor panobinostat sensitizes gastric cancer cells to anthracyclines via the induction of *CITED2* [54]. Moreover, the drug response of multiple small molecules exhibited strong drug resistance to the KD gene *SPOCK1* (response effects from 0.47 to 0.59), while several small molecules showed strong drug sensitivity to the KD gene *PFAS* (response effects from 0.48 to 0.51) (Fig. 5a, e). In addition, we further evaluated whether the KD genes could influence drug responses across 23 gastric cancer cell lines from the Cancer Genome Project (CGP). Indeed, the drug response patterns of several KD genes (such as *SPCK1* and *PFAS*) in CGP were similar to those in CCLE (Fig. S3). Notably, the drug response patterns of multiple KD genes, including *NAA15*, *RNF139*, and *ETFD*, were complementary in two cell line models (Fig. 5e, Fig. S3). This result suggests that it is necessary to use a combination of two cell line models to explore drug responses [55]. In summary, we used cellular models to study the drug response mechanisms of KD genes on the transcriptional level. The effect of small molecule compounds on KD genes can guide researchers in new drug research and development, and has potential application value.

### Clinical application of KD gene signatures in gastric cancer patients

**Fig. 6:**
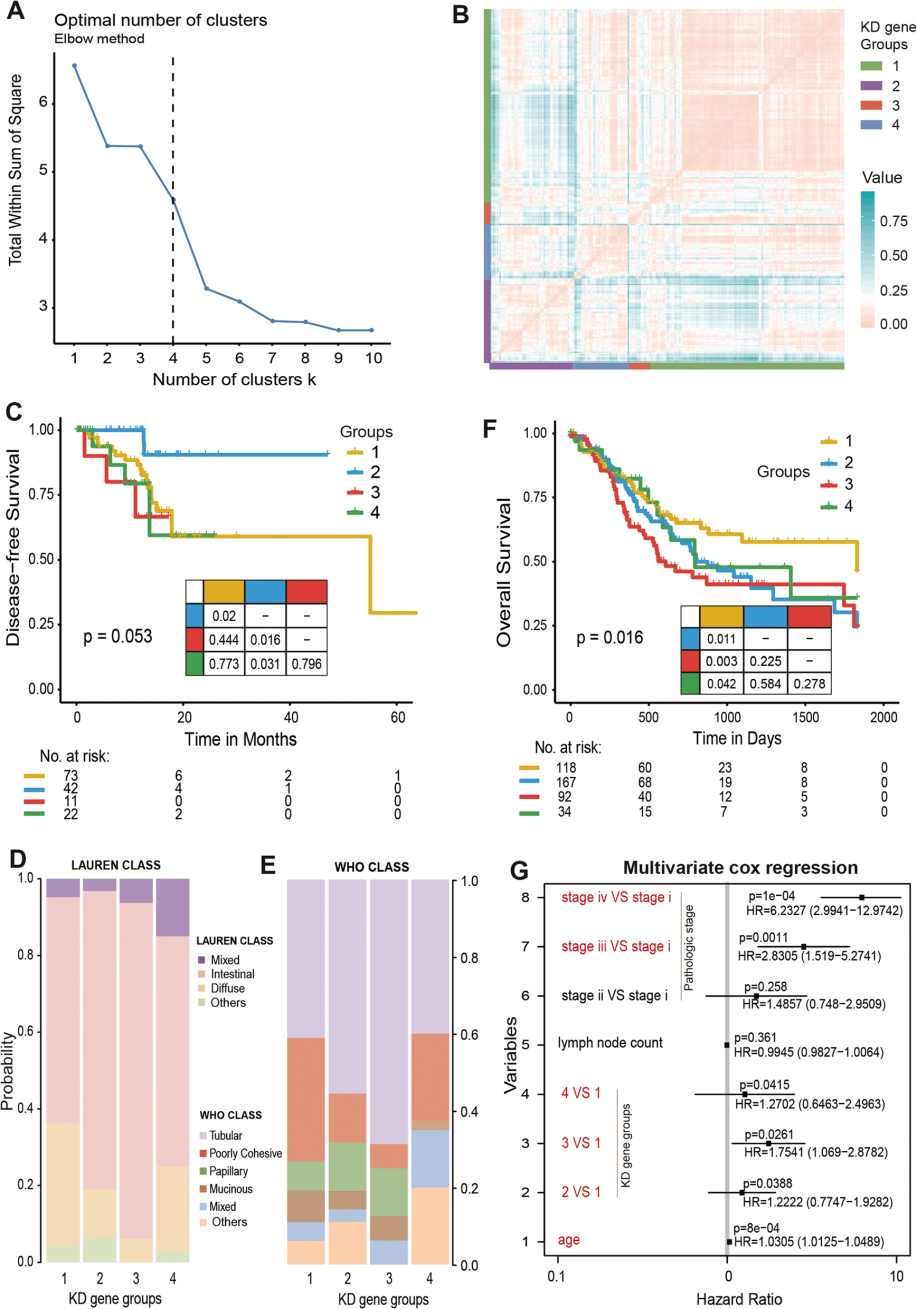
Prognostic efficacy of KD gene signatures. **a**, Determine the optimal number of clusters in hierarchical clustering using Elbow method. **b**, Clustering analysis were performed based on the expression level of KD genes, which showed pearson dissimilarity between patients with gastric cancer. **c**, The KM curve shows disease-free survival (DFS) time of patients across all groups, log-rank test. **d**, **e**, The proportion of patient groups of KD gene signatures in clinical features, including LAUREN (**d**) and WHO class (**e**). **f**, The KM curve shows the overall survival (OS) time of patients in extra data. **g**, Multivariate COX regression analysis (corrected tumour stage, lymph node count, and age). Red means *p* < =0.05

In the above results, we identified 31 prognosis-related KD genes (Supplementary Table 1). To explore the global clinical application value of all KD genes, we constructed sample grouping signatures based on the transcriptional level of the KD genes and verified them in multiple sets of data. In brief, we grouped patients based on the expression of the KD genes using the hierarchical clustering method. We found that when 265 GC patients were clustered into 4 groups, the model performed the best in terms of classification (Fig. 6a). There were 146, 63, 16 and 40 patients in groups 1–4, respectively (Fig. 6b). By calculating the Pearson dissimilarity between the samples, we found that the distances within each group were relatively close, and the distances between the groups were far, which were in line with our findings (Fig. 6b).

To reveal the clinical benefits of the KD gene signature in GC, the log-rank test was used to explore the survival outcomes between patient groups. The KM curve showed that the patients in group 2 had the longest DFS time (Fig. 6c). By observing the patient’s genomic variation events, we found that the patients in group 2 mainly carried variations in *KIAA0196* (also known as *WASHC5*), *EIF3H*, *MRPL13*, *MTSS1*, *DERL1*, and *RNF139* (Fig. S4A). Compared with the patients in group 2, the patients in group 1 presented significantly worse DFS (*P* = 0.02, log-rank test; Fig. 6c), and these patients mainly carried genomic events in *TRMT12*, *TRPA1*, *TCEB1*, *MRPS28*, *PON2* and *SHFM1* (Fig. S4A). Additionally, both group 3 and group 4 patients showed significantly worse DFS (Fig. 6c). However, the four KD gene signatures did not show significant prognostic efficacy for the patients’ overall survival (OS) time (Fig. S6A). In addition, we found that group 3, with the worst survival, showed a higher proportion of the Lauren intestinal class (proportion 87.5%) and WHO tubular class (68.8%) (Fig. 6d, e). The other three groups all showed a lower proportion of the Lauren intestinal class (58.9, 77.8, and 60.0% for group 1, group 2, and group 4, respectively) and WHO tubular class (41.1, 55.6, and 40.0% for group 1, group 2, and group 4, respectively). In contrast, group 3 showed a lower percentage of the Lauren diffuse class and WHO poorly cohesive class (both were 6.25%) (Fig. 6d, e). We also assessed other clinical features of GCs between these groups and found that the patients in group 3 were older in age, had fewer tumour cells, and showed more tumour lymphatic infiltration, however, these features did not show statistical significance (Fig. S5).

To further elucidate the clinical value of our KD genes in GC, we used extra datasets for validation. Similarly, through the expression levels of the KD genes, we subtyped 443 patients into 4 groups based on hierarchical clustering (Fig. S6BC). The KM curve indicated that these four groups of KD gene signatures had significant prognostic efficacy for patients’ overall survival (OS) time (*P* = 0.016, log-rank test; Fig. 6f). Compared with group 1, the patients within the other three groups showed significantly worse OS times (*P* = 0.011, 0.003 and 0.042 for group 2, group 3 and group 4, respectively). Using a multivariate Cox proportional hazards model to adjust for some clinical features, including age, tumour stage, and number of lymph nodes, we found that the other three groups were risk factors compared to group 1 (group 2 (HR = 1.22, 95% CI [0.77–1.92], *p* = 0.039), group 3 (HR = 1.75, 95% CI [1.07–2.88], *p* = 0.026), and group 4 (HR = 1.27, 95% CI [0.65–2.50], *p* = 0.042) compared to the patients within group 1, respectively; Fig. 6g). This finding shows that the signatures of our KD genes were independent prognostic factors. The Cox model also showed that compared to tumour stage I, both stage III (HR = 2.83, 95% CI [1.52–5.27], *p* = 0.001) and stage IV (HR = 6.23, 95% CI [2.99–12.97], *p* = 1e-04) were risk factors (Fig. 6g). In addition, patients’ age showed a correlation with the patients’ OS time (HR = 1.03, 95% CI [1.01–1.05], *p* = 8e-04). Similarly, our results suggest that in group 3, which had a poorer OS, there was a higher proportion of patients who were enriched in the more malignant tubular-intestinal type subclass (Fig. S6D), and more advanced TNM stages T3 and N2 (Fig. S6E). In summary, we used KD genes to construct the group signatures for gastric cancer patients and validated their association with patient survival in multiple datasets, which suggested that the KD genes we identified can be used as prognostic biomarkers for further study by basic experimental and clinical researchers.

## Discussion

To date, although many bioinformatics tools dedicated to driver mutation identification have been developed [13, 14], distinguishing driver mutations from passenger mutations poses a formidable challenge [15]. Therefore, it is urgent to identify cancer drivers and understand them at the functional level. In this study, we integrated multi-omics data to identify the cancer drivers and their dysregulation factors in patients with gastric cancer. After analysing the data of 293 patients from TCGA, we identified 31 prognosis-related key driver (KD) genes. Utilizing functional enrichment analysis of the KD genes, we characterized their affected cancer hallmarks and their related biological functions, such as programmed cell death and antigen processes. The drug response pattern and transcriptional signatures of the KD genes reflect their clinical application value. Combining DNA copy number alterations and mutations can help us avoid the limitations of traditionally identifying driver events. This study proved that the integration of multi-omics data enables the discovery of novel driver molecules and their dysregulation mechanisms during tumourigenesis.

Cancer genes generally induce dysregulation by their regulators and exert driver roles in cancer. Based on the regulatory relationships between regulators and target genes [23], we constructed a transcriptional dysregulation network of KD genes (Fig. 3d). The dysregulation factors of KD genes play a crucial role in cell growth and development [34, 35]. In our study, the KD gene *SPOCK1* was regulated by miR-155-5p, which can form a regulatory feedback loop with *STAT1* and might trigger cancer immunoediting to allow tumour cells to escape immunosurveillance and even to promote tumourigenesis [37]. In addition, the transcription of the KD gene *ETFDH* was upregulated by the TF *SREBF1.* Although the correlation coefficients between these regulators and KD genes were not very strong (0.2–0.4), which may be due to subtle differences in data standardization, the correlation was significant and not accidental. Previous studies have shown that *SREBF1* is a key regulator of fatty acid metabolism and plays a pivotal role in the transcriptional regulation of different lipogenic genes that mediate lipid synthesis, thus acting as a cancer promoter in human diseases [43, 44]. Our study also showed that both miRNAs and TFs play a hub regulatory role in the dysregulation of KD genes, which suggests that dysregulation factors play a crucial role in the process of biological metabolism. Therefore, studying these dysregulation factors may facilitate the discovery of biomarkers.

During the process of genomic variation and natural selection, several driver events exhibited different combination mutational patterns to drive cancer formation, and formed evolutionary dependence. These evolutionary dependence drivers are always highly functionally associated, such as participating in similar biological processes, mediating pathway crosstalk and cooperatively promoting clonal expansion or selective sweep [29, 56]. The functional enrichment analysis results showed that some KD genes were enriched in apoptosis-associated (such as “Programmed cell death” and “Apoptosis-related network due to altered Notch3 in cancer”) and immune-associated functions (such as “Antigen processing: Ubiquitination & Proteasome degradation”), which were related to the corresponding cancer hallmarks, “Evading Apoptosis”, “Evading Immune Detection” and “Tumour Promoting Inflammation”, respectively. In addition, these KD genes offer new insights into molecular mechanisms and have novel prognostic and drug response potential for clinical practice. These findings suggested that the KD genes and dysregulated factors we identified by integrating multi-omics data could have important implications for understanding cancer evolution as well as for diagnostic and therapeutic approaches; thus, they might play crucial roles and are worthy of further exploration.

## Conclusions

This study integrated multi-omics data to discover novel driver molecules and their dysregulation mechanisms based on copy number alteration, somatic mutation, and transcription level analysis. We revealed the clinical application value of KD genes through drug response patterns and transcriptional signatures. As a next step, based on our research results, clinicians and biological experimenters can further confirm the function of these KD genes and their pathogenic mechanism through experiments. Cell models, animal models, and preclinical experiments should be performed to verify the clinical effects of the KD genes, such as targeted therapy. Thus, the conclusions of our study may be translated into clinical applications. These results will pave the way towards understanding the potential mechanisms that govern GC progression, which will be useful in clinical practice and might prompt the development of novel therapeutic targets for GC patients.

## Supplementary Information

**Additional file 1: **Contains the supplementary figures. **Figure S1.** Hierarchical clustering map shows two expression profile datasets of gastric cancer from the GEO database. **Figure S2.** The Pearson correlations between the KD genes and their dysregulation factors on expression level. **Figure S3.** The drug response pattern of the drugs in all KD genes in the Cancer Genome Project (CGP) cell model. **Figure S4.** KD genes carried in gastric cancer patients in two datasets. **Figure S5.** Distribution of clinical features in patient groups. **Figure S6.** Prognostic efficacy of KD gene signatures.

**Additional file 2.** Contains KD genes and their regulators. The gene name and associated TFs and miRNAs are reported.

**Additional file 3.** Contains 4 TFs and 5 miRNAs were confirmed by enrichment.

## Data Availability

The datasets used and analysed during the current study are available in the cBioPortal (https://www.cbioportal.org/), GEO (https://www.ncbi.nlm.nih.gov/geo/), Transfac (http://genexplain.com/transfac/), UCSC (https://genome.ucsc.edu/), Chipbase (http://rna.sysu.edu.cn/chipbase/), miTarbase (http://mirtarbase.cuhk.edu.cn/php/index.php), Starbase (http://starbase.sysu.edu.cn/) and Firehose (https://gdac.broadinstitute.org/) repository.

## Abbreviations

GC: Gastric cancer
CNA: Copy number alteration
OS: Overall survival
DFS: Disease-free survival
KD genes: Key driver genes
TF: Transcriptional factor
GEO: Gene expression omnibus
MF: Molecular function
BP: Biological process
CC: Cellular component
CCLE: Cancer cell line encyclopedia
CGP: Cancer genome project
WSS: Within-cluster sum of square
MIF: Migration inhibitory factor
