# Supplementary material for: Identification of novel prognostic biomarkers by integrating multi-omics data in gastric cancer
Source: BMC Cancer. 2021 Apr 26;21:460. doi: 10.1186/s12885-021-08210-y (PMC8073914; doi:10.1186/s12885-021-08210-y)
Supplement: Supplementary file 1 — Additional file 1: Contains the supplementary figures. Figure S1. Hierarchical clustering map shows two expression profile datasets of gastric cancer from the GEO database. Figure S2. The Pearson correlations between the KD genes and their dysregulation factors on expression level. Figure S3. The drug response pattern of the drugs in all KD genes in the Cancer Genome Project (CGP) cell model. Figure S4. KD genes carried in gastric cancer patients in two datasets. Figure S5. Distribution of clinical features in patient groups. Figure S6. Prognostic efficacy of KD gene signatures. [file 12885_2021_8210_MOESM1_ESM.docx]

*Supplementary figures*


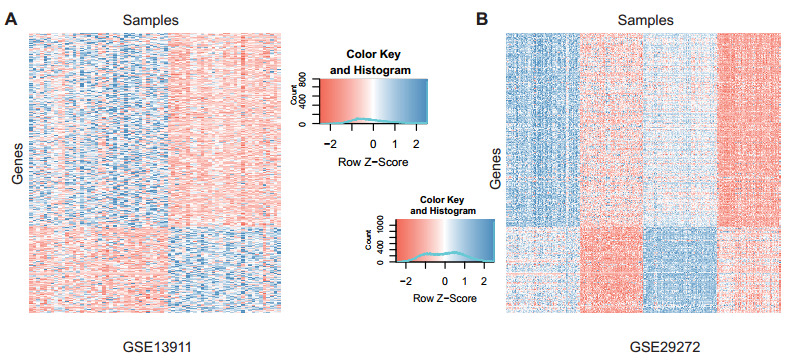


**Figure S1.** Hierarchical clustering map shows two expression profile datasets of gastric cancer from the GEO database. The accession code were GSE13911 (**A**) and GSE29272 (**B**).


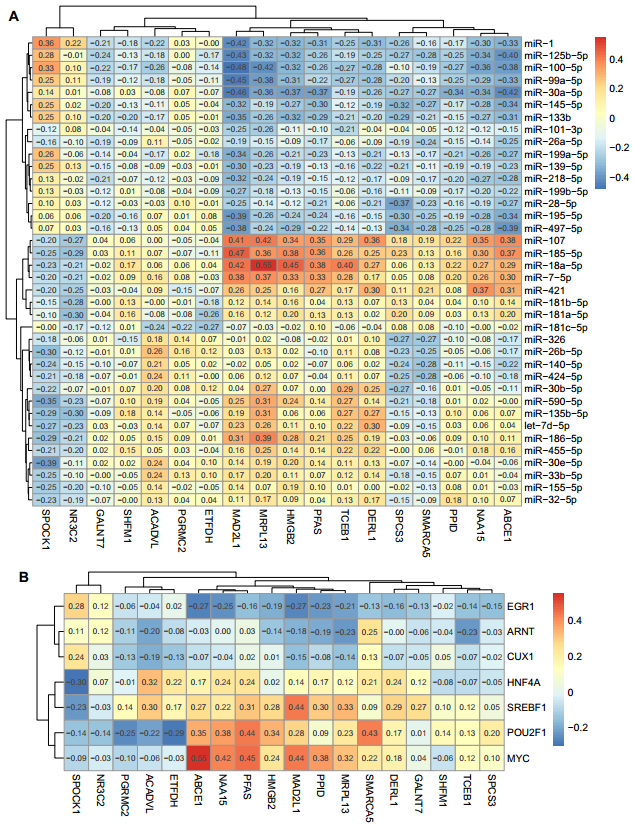


**Figure S2. The** Pearson correlations between the KD genes and their dysregulatory factors (**A**, miRNAs; **B**, TFs) on expression level. Red color indicates positive correlation, blue color indicates negative correlation, and the numerical value indicates the Pearson's correlation coefficient.


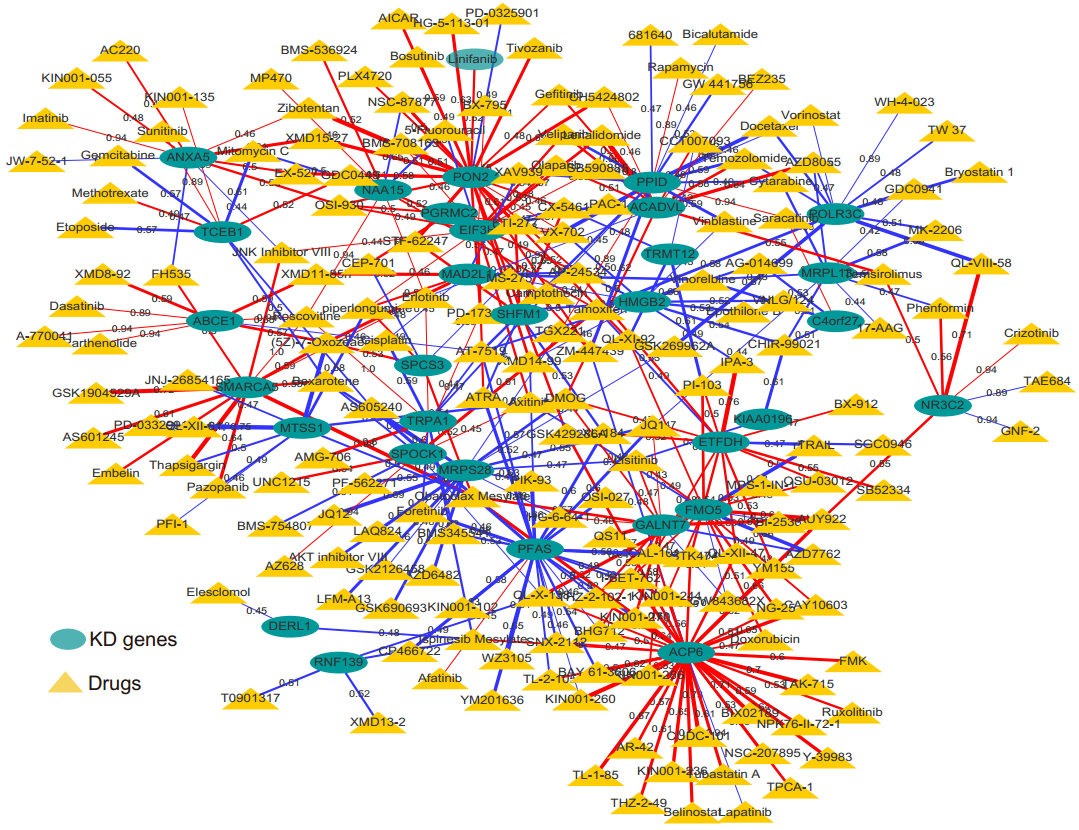


**Figure S3.** The drug response pattern of the drugs in all KD genes in the Cancer Genome Project (CGP) cell model at the Wellcome Trust Sanger Institute, including drug resistance (red) and sensitivity (blue), and line thickness indicates response effect.


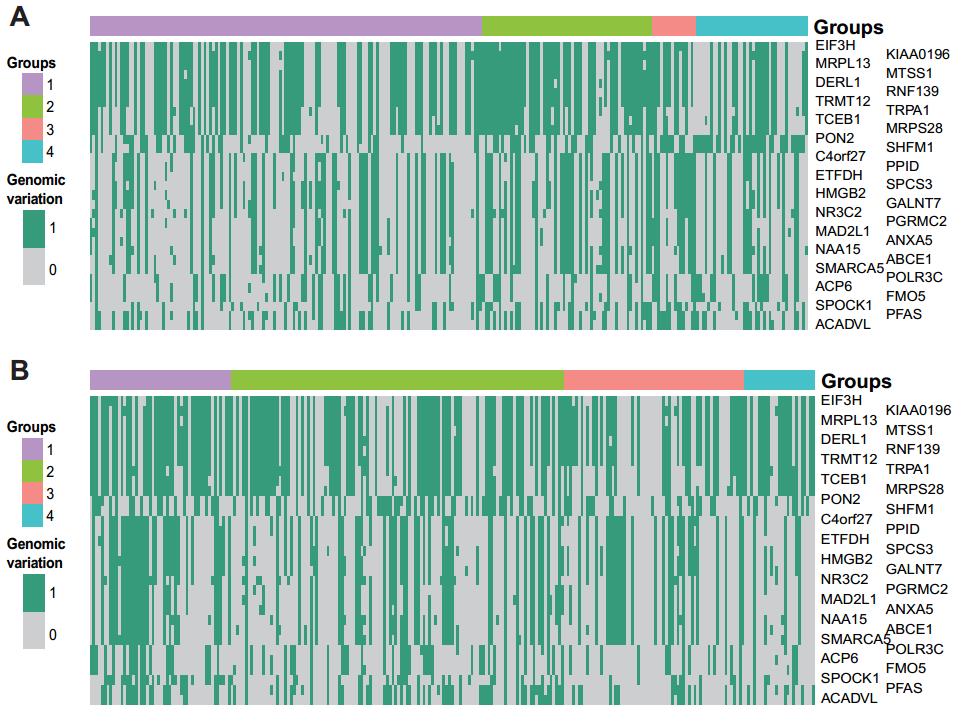


**Figure S4. KD genes carried in gastric cancer patients in two data sets.** TCGA gastric cancer datasets from cBioPort data sources (**A**), and extra datasets for gastric cancer patients from the Firhose database (**B**). The patient groups were constructed by KD gene signatures.


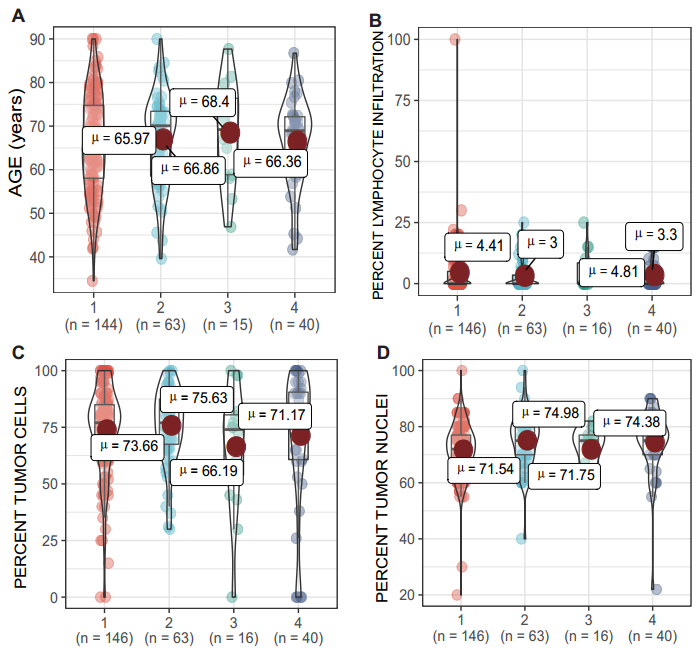


**Figure S5. Distribution of clinical features in patient groups.** The clinical features included age (**A**), tumor lymphatic infiltration (**B**), tumor cells (**C**), and tumor nuclei (**D**).


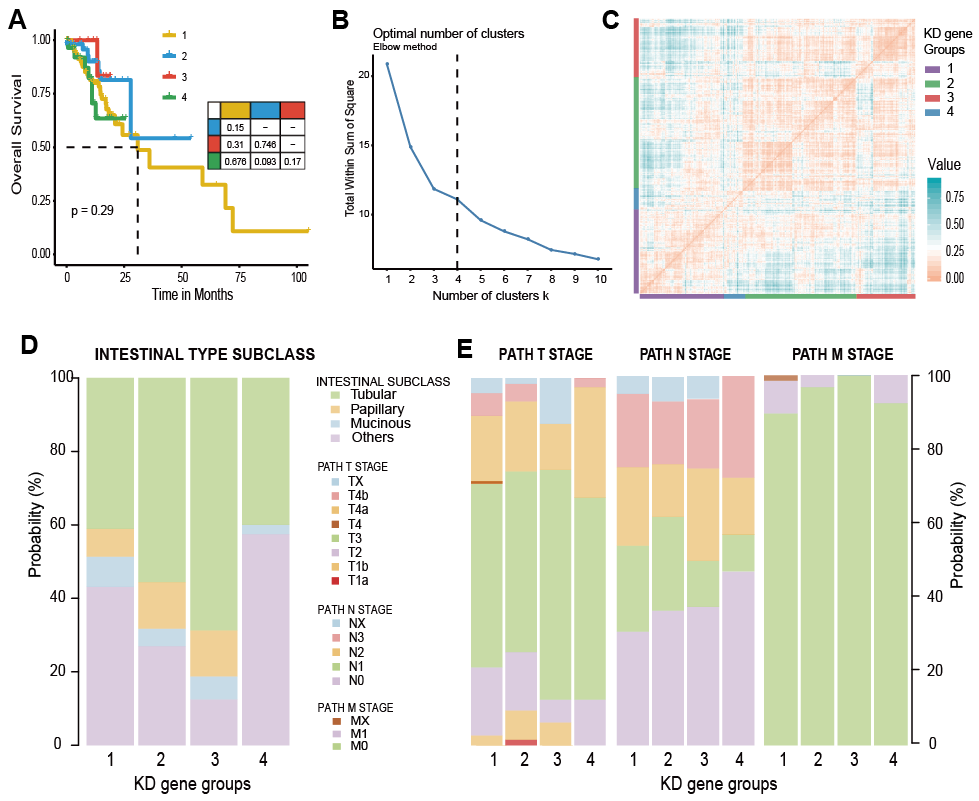


**Figure S6. Prognostic efficacy of KD gene signatures** (Corresponding to Figure 6 in the main text)**. A**, The KM curve shows the overall-survival (OS) time of gastric cancer patients from cBioPort. **BC**, Clustering analysis based on the expression level of KD genes in extra datasets. **DE**, The proportion of the patient groups in clinical features, including INTESTINAL TYPE subclass (**D**) and TNM stages (**E**).
